# Associations between Determinants of Food Choice and the Healthy Eating Index-2020 in Adults: An NHANES 2017–March 2020 Analysis

**DOI:** 10.1016/j.cdnut.2026.109395

**Published:** 2026-06-12

**Authors:** Kayla E Tate, Kristina S Petersen

**Affiliations:** Department of Nutritional Sciences, Pennsylvania State University, University Park, PA, United States

**Keywords:** diet quality, food choice determinants, Healthy Eating Index, HEI-2020, NHANES

## Abstract

**Background:**

Strategies for improving suboptimal diet quality are needed and may be informed by investigating associations between determinants of food choice and diet quality.

**Objectives:**

This study aims to assess associations between determinants of food choice and diet quality assessed by Healthy Eating Index-2020 (HEI-2020) in a nationally representative sample of United States adults.

**Methods:**

This cross-sectional analysis included adult participants in the NHANES 2017–March 2020. Determinants of food choice were identified using a previously proposed conceptual framework and included modifiable (i.e., use of nutrition labels, consumption of food away from home, familiarity with MyPlate, perceived diet quality, typical work schedule, number of hours worked per week) and nonmodifiable (i.e., age, sex, race, BMI, education, relationship status) determinants. Survey-weighted univariate and multivariate hierarchical regression was used to assess the relationship between food choice determinants and the HEI-2020 score. Significant variables from the final model were used to predict adequacy and moderation component subscores of the HEI-2020.

**Results:**

In the final model, modifiable determinants of diet quality associated with the HEI-2020 score included use of nutrition labels, frequency of food away from home, and perceived diet quality (*R*^*2*^_adj_ = 0.23, *P* < 0.001). Those reporting rarely or never using nutrition labels had a 6.73-point lower HEI-2020 score than those reporting using them always or most of the time (*P* < 0.001). Those consuming ≥5 meals away from home in the past week had a 4.78-point lower HEI-2020 score than those consuming 0 meals away from home per week (*P* = 0.004). Those reporting fair or poor perceived diet quality had a 5.15-point lower HEI-2020 score relative to those with excellent or very good perceived diet quality (*P* < 0.001). Determinants accounted for a higher proportion of variance in the adequacy score (*R*^*2*^_*a*dj_ = 0.26, *P* < 0.001) than the moderation score (*R*^*2*^_adj_ = 0.09, *P* < 0.001).

**Conclusions:**

These findings suggest that interventions seeking to meaningfully improve diet quality may benefit from simultaneously targeting multiple determinants of food choice.

## Introduction

Cardiometabolic diseases (CMDs), including cardiovascular disease, type 2 diabetes, and cerebrovascular diseases, are leading causes of disability and mortality in the United States [1]. Suboptimal diet quality is the leading modifiable risk factor for CMD development [2]. High-quality dietary patterns, characterized by adequate intake of vegetables, fruits, whole grains, nuts, seeds, and legumes, and limited intake of refined grains, saturated fats, added sugars, and sodium, reduce risk of CMDs [3,4]. Diet quality is suboptimal across every race, ethnicity, age group, and socioeconomic status group in the United States [5]. Thus, identifying modifiable factors associated with diet quality may assist with developing targeted interventions to reduce risk of CMDs.

Determinants of food choice are complex and influenced by a wide variety of factors ranging from societal to individual-level influences [6]. Societal-level influences include factors such as agricultural policies and food availability [7], whereas individual-level influences include taste preferences [8] and hunger [9]. Chen and Antonelli’s multidisciplinary conceptual framework of food choice determinants classifies determinants into 5 broad categories, including: *1*) food-internal factors; *2*) food-external factors; *3*) personal-state factors; *4*) cognitive factors; and *5*) sociocultural factors [10]. Food-internal factors include sensory and perceptual properties of foods (e.g., flavor, smell, texture, perceived nutritional value), whereas food-external factors include the information present and the social and physical context where food choices are made (e.g., nutrition labels, social norms, eating location). Personal state and cognitive factors can respectively be defined as the biological, physical, psychological, cognitive, and social factors that differ between individuals. Examples of personal-state factors include physiological hunger, whereas examples of cognitive factors include the knowledge, skills, attitudes, and personal identities related to food choices. Sociocultural factors can be defined as macrolevel factors that influence food choice and shape food environments (e.g., culture, economic variables, political elements).

Much research has investigated associations between single determinants of food choice and diet quality in nationally representative samples. This work shows that consumption of food away from home [11], using nutrition labels [12], perceived diet quality [13], and food insecurity [14] are all associated with diet quality. However, when associations between individual determinants and diet quality are examined in isolation, the proportion of total variance in diet quality predicted by these determinants is small [13]. The small proportion of variance in diet quality predicted by single determinants highlights the importance of simultaneously considering several domains of food choice to best inform interventions. Previous research investigating associations between individual food choice determinants and diet quality has not used a conceptual framework to identify multiple relevant determinants to include in analyses. Thus, there is a literature gap surrounding how multiple determinants of food choice from a comprehensive conceptual framework relate to diet quality in nationally representative samples. The present study aims to assess associations between determinants of food choice identified using Chen and Antonelli’s conceptual framework [10] and diet quality, as assessed by the Healthy Eating Index-2020 (HEI-2020) in a nationally representative sample of United States adults. This work is expected to assist with identifying intervention targets for improving diet quality and reducing CMDs.

## Methods

### Study overview

The present study was a cross-sectional analysis of NHANES 2017–March 2020. This wave was chosen because it is the most recent publicly available sample with an available food patterns equivalents database. Additional waves were not included because of inconsistencies in the information collected on food choice determinants. Sample collection details are reported elsewhere [15]. Briefly, NHANES is a cross-sectional multistage probability-based nationally representative sample of the civilian noninstitutionalized population in the United States. For this study, survey items were mapped onto Chen and Antonelli’s framework of determinants of food choice [10] and used in regression models to predict the HEI-2020 score.

### Population

The present analysis included adults aged ≥20 y who were not on a special diet, not pregnant or breastfeeding, had complete and reliable 24 h-recall dietary data for 2 d, and had complete data for each independent variable. In addition, only respondents with positive dietary two-day sample weights were included in the analyses consistent with recommendations for analyses involving both days of 24 h-recall data [16].

### Measures

The HEI-2020 is a diet quality index that assesses adherence to the 2020–2025 Dietary Guidelines for Americans [17]. It is calculated based on the intake of 9 adequacy dietary components, including total fruits, whole fruits, total vegetables, greens and beans, whole grains, dairy, total protein foods, seafood and plant protein, and fatty acids, and 4 moderation dietary components, including refined grains, saturated fats, added sugars, and sodium. A value for each intake category is assigned based on prespecified ranges [17], and each component score is summed to derive a total score from 0 to 100, with higher scores indicating higher diet quality. For the present study, the HEI-2020 scoring per-person algorithm was used to calculate an HEI-2020 score for each participant [18]. The scoring algorithm only derives HEI-2020 scores for NHANES participants, with dietary recalls meeting predefined 24 h-recall reliability standards [19,20]. Thus, no participants were excluded based on energy intake. The aggregated adequacy and moderation scores used in the models were calculated by summing the scores for adequacy and moderation components, respectively.

NHANES questionnaires were reviewed for variables to be mapped onto Chen and Antonelli’s [10] determinants of food choice framework, which includes the following 4 domains: *1*) food-external factors; *2*) personal-state factors; *3*) cognitive factors; *4*) society-related features. See Table 1 for a description of framework domains and NHANES variables mapped onto each domain. The purpose of using a conceptual framework of food choice determinants was to identify relevant variables to include in the analysis rather than to draw conclusions regarding the relationship between the framework domains and diet quality. Thus, decisions regarding the specific domain each variable maps onto do not impact the results of the present analyses or their interpretations. To assist with analysis and interpretation, variables identified using the conceptual framework were classified as modifiable or nonmodifiable determinants. Specifically, nonmodifiable determinants were entered in the sequential model-building procedure before modifiable determinants. Modifiable determinants of food choice included use of nutrition facts, perceived diet quality, frequency of consuming food away from home, number of hours typically worked in a week, typical work schedule, familiarity with MyPlate, income-to-poverty ratio (IPR), and food security status. Nonmodifiable determinants of food choice included age, sex, race, BMI, education, and relationship status.

**TABLE 1:** The 5 main factors linked to food choice and NHANES variables mapped onto each domain

Five main factors influencing food choice^1^	Subfactors within the main factor	Subfactor examples	NHANES variables mapped onto the domain
Food-internal factors	Sensory features and perceptual features of foods	Flavor, taste, smell, texture, color, portion size, nutrition and health value, and quality	None
Food-external factors	Information present when food is consumed	Nutrition labels, health claims, packaging, aesthetics, ethics of production history, brand, and advertisement	Use of nutrition labels
	Social environment where food is consumed	Social norms from family, peers, and media, including ethical concern and social context, when food choice is made	
	Physical environment where food is consumed	Availability and accessibility of food products, food retail environment, and time	Hours worked, typical work schedule
Personal-state factors	Biological features	Genetic factors, personal dietary patterns and metabolism, physical condition, such as health	
	Physiological needs	Hunger, appetite, weight status	BMI
	Psychological components	Emotion, motivation, personality	
	Habits and experiences		Frequency of consuming food away from home
Cognitive factors	Knowledge and skills		Familiarity with MyPlate, education
	Attitude, liking, and preference		
	Anticipated consequences		
	Personal identity	Demographic features, such as age, gender, ethnic identity, education, and personal values and beliefs	Perceived diet quality, age, sex, race and ethnicity, marital status
Sociocultural factors	Culture	Norms and values	
	Economic variables	Income, socioeconomic status	Food security status, income-to-poverty ratio
	Political elements	Agricultural and food policy, or other regulations	

1 From Chen and Antonelli’s conceptual model of food choice.

Several variables were recoded before analysis. Use of nutrition labels was condensed into 3 categories, including “always/most of the time”, “sometimes”, and “rarely/never”. Hours worked was categorized into 3 levels representing “part time” (i.e., <35 h a week), “full time” (i.e., 35–40 h a week), and “greater than full time” (i.e., ≥40 h a week). Typical work schedule was condensed into 3 categories, including “traditional 9–5”, “night shifts” (i.e., evenings, nights, early mornings), or “variable schedule”. Frequency of consuming food away from home was condensed into 4 levels, including “0 meals per week”, “1–2 meals per week”, “3–4 meals per week”, and “≥5 meals per week”. Perceived diet quality responses were condensed into 3 categories, including “excellent/very good”, “good”, and “fair/poor”. Familiarity with MyPlate was coded as a binary variable with levels of “yes” and responses of “no” or “don’t know” classified as “no”. Age was categorized into a younger (i.e., 20–50 y) and older (51 y and older) age group. Sex was classified as male or female. BMI was categorized according to standard BMI ranges (i.e., <18.5, 18.5–<25, 25–<30, ≥30 kg/m^2^). Education was categorized into 4 categories: *1*) “<9th grade education” or “some high school”; *2*) “high school graduate”; *3*) “some college or associate’s degree”; and *4*) “college graduate or above”. Relationship status was recoded into categories of: *1*) “married” or “cohabitating”; *2*) “widowed”, “divorced”, or “separated”; *3*) “never married”. IPR was recoded into a binary variable with <1.0 classified as “below the federal poverty line” and ≥1.0 classified as “above the federal poverty line”. IPR was dichotomized to facilitate result interpretation and to align with a policy-relevant threshold for poverty status characterization [21].

### Statistical analysis

Analyses were conducted with SAS version 9.4 using PROC SURVEY REG or PROC SURVEY FREQ, with day 2 dietary intake sample weights used for all analyses. Univariate regression models were used to determine unadjusted associations between identified modifiable and nonmodifiable factors and the HEI-2020 score. Variables significantly associated with the HEI-2020 score were included in the model-building sequence. Model 1 was composed of nonmodifiable variables only, model 2 was nonmodifiable variables and society-level features, model 3 added food-external features to model 2 variables, and model 4 included model 3 variables in addition to personal state and cognitive variables. Significant variables from the final model were used in multivariate regression models predicting aggregated and individual adequacy and moderation component subscores of the HEI-2020. Models were assessed for normality and homoscedasticity of residuals, multicollinearity, and autocorrelation. Because of the complex multistage sampling method used for NHANES, all models violate the randomness assumption, and results should be interpreted with caution. *P* < 0.05 was used for statistical significance.

## Results

There were 15,560 participants in the 2017–2020 NHANES wave. Of these participants, 6328 were excluded due to age <20 y, 2828 were excluded due to reporting adhering to a special diet, 155 were excluded due to pregnancy or breastfeeding, 5 were excluded due to missing HEI-2020 scores, and 3746 were excluded due to missing data for covariates or independent variables. The final analytical sample was 2498. See Table 2 for survey-weighted sample characteristics.

**TABLE 2:** Survey-weighted sample characteristics

Variable	*N* = 2498	Survey-weighted *N* = 97,582,600	Survey-weighted value^1^
HEI-2020 total score^2^			52.12 ± 0.67
Sex			
Male	1302	52,920,238	54.23 (1.99)
Female	1196	44,662,362	45.76 (1.99)
Age	2498	97,582,600	42.24 ± 0.52^2^
20–50 y	1585	69,263,777	70.98 (1.54)
51 y and older	913	28,318,823	29.02 (1.54)
Food security status			
Full food security	1621	71,857,536	73.64 (1.85)
Marginal food security	345	10,941,504	11.21 (1.18)
Low food security	327	9,546,818	9.78 (0.96)
Very low food security	205	5,236,743	5.37 (0.59)
Education			
<12th grade	293	6,456,963	6.62 (0.80)
12th grade graduate	535	23,101,203	23.67 (1.95)
Some college or associates degree	902	31,004,743	31.77 (1.88)
College graduate or above	768	37,019,690	37.93 (3.29)
Race/ethnicity			
Mexican American	540	16,164,448	16.56 (1.77)
Non-Hispanic White	826	60,018,880	61.51 (3.46)
Non-Hispanic Black	706	11,305,766	11.58 (1.77)
Other or Multiracial	426	10,093,505	10.34 (1.23)
Relationship status			
Married or cohabitating	1479	59,410,810	60.88 (1.84)
Separated, widowed, or divorced	432	14,588,104	14.95 (1.16)
Never married	587	23,583,685	24.17 (1.33)
Income-to-poverty ratio (IPR)			
Low income (IPR <1)	353	10,453,176	10.71 (1.10)
Middle/high income (IPR ≥1)	2145	87,129,424	89.29 (1.10)
Use of nutrition labels			
Always/most of the time	891	33,936,737	34.78 (1.47)
Sometimes	933	39,856,400	40.84 (1.57)
Rarely/never	674	23,789,463	24.39 (1.82)
Hours per week worked			
>35	653	25,396,572	26.03 (1.57)
35 to <40	984	32,877,004	33.69 (1.82)
≥40	861	39,309,023	40.28 (2.09)
Typical work schedule			
Traditional 09:00–17:00	958	38,217,691	39.17 (1.92)
Evenings, nights, early mornings	686	23,844,767	24.44 (1.75)
Variable	854	35,520,142	36.40 (1.40)
BMI (kg/m^2^)			29.08 ± 0.26^2^
<18.5	37	1,332,366	1.37 (0.42)
18.5 to <25	638	26,907,435	27.57 (1.68)
25 to <30	782	31,211,690	31.99 (2.03)
≥30	1041	38,131,108	39.08 (2.39)
Frequency of consuming food away from home in the last 7 d			
0 meals	321	9,613,236	9.85 (0.95)
1–2 meals	745	28,675,903	29.39 (1.48)
3–4 meals	619	23,506,042	24.09 (1.16)
≥5 meals	813	35,787,418	36.67 (1.70)
Familiarity with MyPlate			
Heard of MyPlate	636	28,447,841	29.15 (2.38)
Never heard of MyPlate	1862	69,134,758	70.85 (2.38)
Perceived diet quality			
Excellent/very good	625	25,441,156	26.07 (1.83)
Good	979	40,067,743	41.06 (1.89)
Fair/poor	894	32,073,701	32.86 (1.46)

1 Data are presented as frequency (%) unless otherwise stated.

2 Reported as mean ± SE.

In bivariate analyses, the modifiable determinants of food choice including use of the nutrition facts label (F(2,25) = 41.32, *P* < 0.0001), frequency of consuming food away from home (F(3,25) = 20.15, *P* < 0.0001), IPR classification (F(1,25) = 6.95, *P* = 0.014), food security status (F(3,25) = 21.97, *P* < 0.0001), and perceived diet quality (F(2,25) = 76.82, *P* < 0.0001) were statistically significant predictors of the HEI-2020 score (Table 3). Of the nonmodifiable factors, age group (F(1,25) = 17.64, *P <* 0.001), sex (F(1,25) = 11.6, *P* = 0.002), education (F(3,25) = 13.97, *P* < 0.0001), race and ethnicity (F(3,25) = 4.22, *P* = 0.015), and relationship status (F(2,25) = 4.22, *P* = 0.023) were associated with the HEI-2020 score. Number of hours worked per week, typical work schedule, and familiarity with MyPlate were not significantly associated with the HEI-2020 score.

**TABLE 3:** Bivariate and multivariate associations between determinants of food choice and the total HEI 2020 score

Characteristic	Unadjusted	Model 1^1^	Model 2^2^	Model 3^3^	Model 4^4^
	---------------------------*β* (SE)-------------------------				
Age					
20–50 y	–4.40 (1.05)∗∗	–4.26 (0.92)∗∗	–4.12 (0.93)∗∗	–3.80 (0.77)∗∗∗	–3.04 (0.78)∗∗
51 y and older	REF	REF	REF	REF	REF
Sex					
Male	–2.57 (0.75)∗	–1.50 (0.74)	–1.60 (0.74)∗	–1.04 (0.72)	–0.74 (0.73)
Female	REF	REF	REF	REF	REF
Education					
<12th grade	–8.80 (1.81)∗∗∗	–9.10 (2.00)∗∗	–8.28 (2.13)∗∗	–6.20 (2.16)∗	–5.46 (2.13)∗
12th-grade graduate	–8.05 (1.56)∗∗∗	–7.62 (1.43)∗∗∗	–7.15 (1.45)∗∗∗	–5.42 (1.24)∗∗	–4.52 (1.24)∗
Some college/Associate’s degree	–6.95 (1.12)∗∗∗	–6.40 (1.12)∗∗∗	–6.10 (1.17)∗∗∗	–5.11 (1.03)∗∗∗	–4.70 (1.05)∗∗
College graduate or above	REF	REF	REF	REF	REF
Race/ethnicity					
Hispanic	–0.56 (0.93)	2.43 (0.97)∗	2.82 (0.99)∗	2.51 (0.97)∗	2.50 (0.85)∗
Non-Hispanic Black	–1.82 (0.93)	0.60 (0.88)	0.97 (0.96)	0.82 (0.92)	0.78 (0.94)
Other or Multiracial	2.53 (0.94)∗	2.93 (0.97)∗	3.06 (0.96)∗	2.91 (1.08)∗	2.29 (1.10)∗
Non-Hispanic White	REF	REF	REF	REF	REF
BMI category (kg/m^2^)					
<18.5	–7.25 (4.10)	–4.09 (4.29)	–4.54 (4.41)	–5.88 (3.97)	–5.95 (4.28)
25–<30	–1.5 (1.60)	–1.52 (1.43)	–1.72 (1.45)	–2.01 (1.34)	–1.88 (1.30)
≥30	–5.47 (1.39)∗∗	–4.57 (1.23)∗	–4.66 (1.27)∗	–4.85 (1.21)∗∗	–3.71 (1.24)∗
18.5 to <25	REF	REF	REF	REF	REF
Relationship status					
Separated, widowed, divorced	0.22 (1.08)	0.28 (0.84)	0.56 (0.85)	0.16 (0.88)	0.28 (0.83)
Never married	–3.64 (1.26)∗	–2.44 (1.21)	–2.12 (1.22)	–2.28 (1.08)∗	–1.85 (1.06)
Married or cohabitating	REF	REF	REF	REF	REF
Income-to-poverty ratio (IPR)					
Low income (IPR <1)	–3.76 (1.42)∗		–0.05 (1.68)	0.33 (1.60)	0.31 (1.61)
Middle/high income (IPR ≥1)	REF		REF	REF	REF
Food security status					
Very low food security	–7.73 (1.01)∗∗∗		–5.00 (1.02)∗∗∗	–5.11 (0.99)∗∗∗	–4.46 (1.06)∗∗
Low food security	–3.49 (1.02)∗		–1.00 (1.14)	–1.04 (1.24)	–0.68 (1.21)
Marginal food security	–3.59 (1.52)∗		–0.93 (1.56)	–0.85 (1.29)	–1.18 (1.23)
Full food security	REF		REF	REF	REF
Using nutrition facts					
Rarely/never	–10.33 (1.14)∗∗∗			–8.28 (1.06)∗∗∗	–6.73 (1.10)∗∗∗
Sometimes	–5.74 (0.94)∗∗∗			–4.94 (0.86)∗∗∗	–3.70 (1.09)∗
Always/most of the time	REF			REF	REF
Perceived diet quality					
Fair/poor	–11.22 (0.96)∗∗∗				–5.15 (0.95)∗
Good	–6.51 (1.19)∗∗∗				–2.96 (1.14)∗∗∗
Excellent/very good	REF				REF
Frequency of food away from home in the last 7 d					
≥5 meals	–8.19 (1.24)∗∗∗				–4.78 (1.23)∗∗
3–4 meals	–6.10 (1.64)∗				–3.76 (1.62)∗
1–2 meals	–2.99 (1.21)∗				–2.59 (1.03)∗
0 meals	REF				REF
Model F statistic		26.54∗∗∗	48.49∗∗∗	160.48∗∗∗	543.64∗∗∗
Adj *R*^*2*^		0.14	0.14	0.19	0.23

1 Nonmodifiable determinants only (i.e., age, sex, education, race/ethnicity, BMI category, relationship status).

2 Nonmodifiable determinants + sociocultural factors (i.e., IPR, food security status).

3 Nonmodifiable + sociocultural factors + food-external factors (i.e., use of nutrition facts).

4 Nonmodifiable + sociocultural factors + food-external factors + personal-state and cognitive factors (i.e., perceived diet quality, frequency of food away from home in the past 7 d).

∗ *P <* 0.05

∗∗ *P <* 0.001

∗∗∗ *P <* 0.0001.

In the final model (*R*^*2*^adj = 0.23, F(24,25) = 543.64, *P <* 0.0001), food security status (F(3,25) = 6.18, *P* = 0.003), using the nutrition facts label (F(2,25) = 21.75, *P <* 0.0001), perceived diet quality (F(2,25) = 19.24, *P <* 0.0001), frequency of consuming food away from home (F(3,15) = 5.57, *P =* 0.005), age group (F(1,25) = 15.15, *P <* 0.001), education (F(3,25) = 6.66, *P =* 0.002), race and ethnicity (F(3,25) = 4.34, *P =* 0.014), and BMI (F(3,25) = 3.42, *P =* 0.03) remained significant predictors of the HEI-2020 score. Those reporting full food security had a 4.46-point higher HEI-2020 score relative to those with very low food security. Those reporting using nutrition labels “rarely/never” had a 6.73-point lower HEI-2020 score relative to those reporting using nutrition labels “always/most of the time”. For perceived diet quality, those who perceived their diets to be “fair/poor” had a 5.15-point lower HEI-2020 score relative to those perceiving their diets to be “excellent/very good”. Consuming 5 or more meals away from home in the last 7 d was associated with a 4.78-point lower HEI-2020 score relative to those who reported consuming 0 meals away from home. Younger adults had a 3.04-point lower HEI-2020 score than older adults, and those with the least education had a 5.46-point lower HEI-2020 score compared with those with the most education. Individuals identifying as Mexican American or other Hispanic (2.50 points), or those identifying as multiracial, Asian, or another race or ethnicity (2.28 points), had higher HEI-2020 scores than those identifying as non-Hispanic White. Individuals with a BMI corresponding to underweight (–5.95 points), overweight (–1.88 points), or obesity (–3.71 points) had lower HEI-2020 scores than those with a normal weight BMI. Sex, relationship status, and IPR were not significant predictors of the HEI-2020 score in the final model.

**FIGURE 1:**
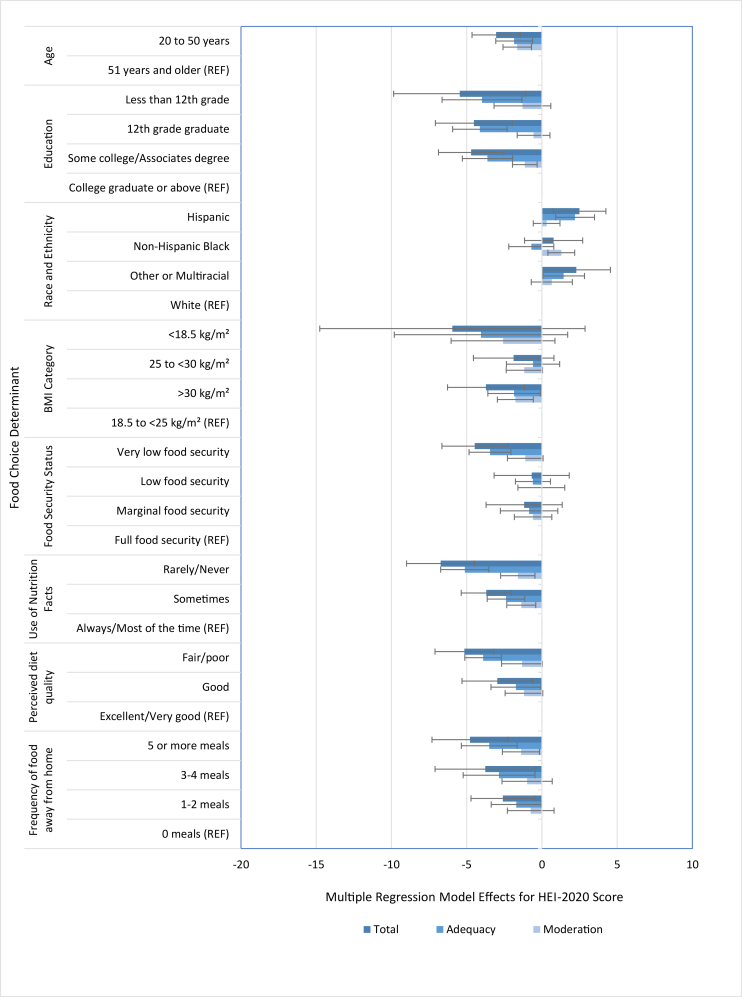
Regression model effects and 95% confidence intervals for total, adequacy, and moderation HEI-2020 scores. This figure is showing multiple regression model betas and 95% confidence intervals from 3 multiple regression models where total, adequacy, and moderation HEI-2020 scores were the outcome variables and modifiable (i.e., frequency of food away from home, perceived diet quality, use of nutrition facts, food security status) and nonmodifiable (i.e., included age, race and ethnicity, BMI category, education) food choice determinants were the predictor variables. HEI-2020, Healthy Eating Index 2020.

Significant variables in the final model predicted more variance in aggregated adequacy HEI-2020 score components (*R*^*2*^adj = 0.26, F(20,25) = 549.78, *P <* 0.0001) than in aggregated moderation components (*R*^*2*^adj = 0.09, F(20,25) = 49.14, *P <* 0.0001). In the adequacy score model, food security status (F(3,25) = 10.40, *P =* 0.001), using the nutrition facts label (F(2,25) = 23.37, *P <* 0.0001), perceived diet quality (F(2,25) = 36.33, *P <* 0.0001), frequency of food away from home (F(3,25) = 7.88, *P =* 0.001), age group (F(1,25) = 9.71, *P =* 0.005), level of education (F(3,25) = 7.61, *P =* 0.001), and race and ethnicity (F(3,25) = 7.55, *P =* 0.001) were all significantly associated with the adequacy score (Figure 1). BMI category was not significantly associated with the adequacy score. In the moderation score model, using the nutrition facts label (F(2,25) = 5.43, *P =* 0.01), age (F(1,25) = 12.82, *P =* 0.001), level of education (F(3,25) = 3.59, *P =* 0.028), and BMI category (F(3,25) = 3.15, *P =* 0.04) were significantly associated with the moderation score. Frequency of food away from home, perceived diet quality, and food security status were not associated with the moderation score.

Significant variables from model 4 were entered into models predicting individual HEI-2020 adequacy and moderation component scores. Of the adequacy components (Supplemental Table 1), total vegetables (*R*^*2*^adj = 0.13, F(20,25) = 36.95, *P <* 0.0001) was significantly associated with education (F(3,25) = 9.04, *P =* 0.0003), race and ethnicity (F(3,25) = 6.87, *P =* 0.002), food security status (F(3,25) = 5.11, *P =* 0.007), using nutrition facts (F(2,25) = 10.89, *P =* 0.001), and perceived diet quality (F(2,25) = 7.92, *P =* 0.002). Greens and beans (*R*^*2*^adj = 0.14, F(20,25) = 118.1, *P <* 0.0001) was significantly associated with education (F(3,25) = 10.92, *P <* 0.0001), race and ethnicity (F(3,25) = 8.09, *P =* 0.001), using nutrition facts (F(2,25) = 7.19, *P =* 0.003), perceived diet quality (F(2,25) = 5.63, *P =* 0.01), and frequency of food away from home (F(3,25) = 7.88, *P =* 0.001). Total fruit (*R*^*2*^adj = 0.14, F(20,25) = 101.76, *P <* 0.0001) was significantly associated with age (F(1,25) = 18.66, *P =* 0.0002), education (F(3,25) = 4.66, *P =* 0.010), race and ethnicity (F(3,25) = 8.7, *P =* 0.001), BMI category (F(3,25) = 3.95, *P =* 0.02), food security status (F(3,25) = 3.08, *P =* 0.046), using nutrition facts (F(2,25) = 9.51, *P =* 0.001), perceived diet quality (F(2,25) = 4.15, *P =* 0.028), and frequency of consuming food away from home (F(3,25) = 6.31, *P =* 0.003). Whole fruits (*R*^*2*^adj = 0.17, F(20,25) = 173.97, *P <* 0.0001) were significantly associated with age (F(1,25) = 36.74, *P <* 0.0001), race and ethnicity (F(3,25) = 6.32, *P =* 0.002), BMI category (F(3,25) = 5.01, *P =* 0.007), food security status (F(3,25) = 6.83, *P =* 0.002), using nutrition facts (F(2,25) = 14.07, *P <* 0.0001), perceived diet quality (F(3,25) = 6.92, *P =* 0.004), and frequency of food away from home (F(3,25) = 5.04, *P =* 0.007). Whole grains (*R*^*2*^adj = 0.12, F(20,25) = 97.35, *P <* 0.0001) were significantly associated with race and ethnicity (F(3,25) = 10.93, *P <* 0.0001), using nutrition facts (F(2,25) = 19.91, *P <* 0.0001), and frequency of food away from home (F(3,25) = 11.41, *P <* 0.0001). Total dairy (*R*^*2*^adj = 0.06, F(20,25) = 27.65, *P <* 0.0001) was significantly associated with age (F(1,25) = 4.6, *P =* 0.042) and race and ethnicity (F(3,25) = 38.98, *P <* 0.0001). Total protein (*R*^*2*^adj = 0.03, F(20,25) = 19.26, *P <* 0.0001) was significantly associated with race and ethnicity (F(3,25) = 5.34, *P =* 0.006), and frequency of food away from home (F(3,25) = 4.93, *P =* 0.008). Seafood and plant protein (*R*^*2*^adj = 0.09, F(20,25) = 31.73, *P <* 0.0001) was significantly associated with age (F(1,25) = 5.97, *P =* 0.022), education (F(3,25) = 6.29 *P =* 0.003), race and ethnicity (F(3,25) = 6.77, *P =* 0.002), perceived diet quality (F(2,25) = 9.93, *P =* 0.001), and frequency of food away from home (F(3,25) = 4.08, *P =* 0.017). Fatty acids (*R*^*2*^adj = 0.05, F(20,25) = 19.91, *P <* 0.0001) were associated with race and ethnicity (F(3,25) = 15.98, *P <* 0.0001) and food security status (F(3,25) = 4.22, *P =* 0.015).

Of the moderation components (Supplemental Table 2), sodium (*R*^*2*^adj = 0.04, F(20,25) = 12.59, *P <* 0.0001) was associated with age (F(1,25) = 15.75, *P =* 0.001) and education (F(3,25) = 3.41, *P =* 0.033). Refined grain (*R*^*2*^adj = 0.08, F(20,25) = 23.38, *P <* 0.0001) was associated with age (F(1,25) = 11.14, *P =* 0.003), race and ethnicity (F(3,25) = 20.23, *P <* 0.0001), and perceived diet quality (F(2,25) = 5.81, *P =* 0.009). Saturated fat (*R*^*2*^adj = 0.05, F(20,25) = 25.93, *P <* 0.0001) was associated with race/ethnicity (F(3,25) = 14.47, *P <* 0.0001) and food security status (F(3,25) = 4.01, *P =* 0.019). Added sugars (*R*^*2*^adj = 0.14, F(20,25) = 684.26, *P <* 0.0001) were associated with education (F(3,25) = 11.18, *P <* 0.0001), race and ethnicity (F(3,25) = 5.01, *P =* 0.007), food security (F(3,25) = 8.35, *P =* 0.001), using nutrition facts (F(2,25) = 24.65, *P <* 0.0001), perceived diet quality (F(2,25) = 5.9, *P =* 0.008), and frequency of food away from home (F(3,25) = 3.08, *P =* 0.046).

## Discussion

The present study investigated associations between determinants of food choice based on Chen and Antonelli’s [10] conceptual framework and diet quality as assessed by the HEI-2020. Of the modifiable determinants, it was found that using the nutrition facts label more frequently, having a higher perceived diet quality, and eating fewer meals away from home were all associated with a higher HEI-2020 score relative to not using nutrition facts, having a lower perceived diet quality, or eating more meals away from home, respectively. Of the nonmodifiable determinants, it was found that having more education, being older, and identifying as Hispanic or multiracial were associated with higher HEI-2020 scores relative to having less education, being younger, or identifying as White, respectively. Lastly, it was found that the final model containing individual-level determinants of diet quality accounted for 23% of the variance in the HEI-2020 score, and the final model variables predicted a larger proportion of the variance in the adequacy components of diet quality than the moderation components.

The findings that the modifiable determinants of diet quality, including using nutrition labels, perceived diet quality, consumption of food away from home, and food security, were associated with diet quality are consistent with previous research. Specifically, in previous waves of NHANES, using the nutrition facts label to make food choices was associated with a modestly higher HEI-2010 score (+4.01 points) relative to not using the nutrition facts labels, [12] which is similar to the results observed in the present analyses (+6.73 points). Regarding perceived diet quality, the finding that higher perceived diet quality predicted higher HEI-2020 scores is consistent with previous literature. However, although there is a positive relationship between measured and perceived diet quality [22], perceived diet quality is often overestimated in those with higher measured diet quality [23]. There is concordance between low perceived diet quality and measured diet quality [24], which is consistent with the finding that those with the lowest perceived diet quality had lower measured diet quality (i.e., ∼5 points) in the present study. Increased consumption of food away from home is associated with poorer diet quality in multiple United States populations, including adults living in Puerto Rico and adults included in previous waves of NHANES [11,25]. In terms of food security status, it is well-established that those with higher food security status typically have higher diet quality than those with lower food security status [26]. Taken together, the findings that using nutrition labels, consuming food away from home less frequently, and having food security are associated with modestly higher diet quality confirm these determinants as potential targets for interventions aiming to improve diet quality.

Nonmodifiable determinants, including age group, race and ethnicity, level of education, BMI, marital status, sex, and IPR, were significantly associated with diet quality in bivariate analyses, but not all remained significant in the final model. Specifically, age group, race and ethnicity, level of education, and BMI remained significant in the final model, whereas sex, IPR, and relationship status did not. The findings that age group [27], race and ethnicity [28], level of education [29], and BMI [30] are independent predictors of diet quality are consistent with the literature. In addition, when considering marital status as a domain of social support, it is consistent with the literature that when other determinants of diet quality are accounted for, including age, sex, race and ethnicity, education, and income, social support is not significantly associated with diet quality [31]. Conversely, it is inconsistent that sex and IPR were not found to be independently associated with diet quality. In previous waves of NHANES, males typically have significantly lower diet quality than females [32]. Coupled with the present findings, this suggests that the relationship between diet quality and sex may be explained through other determinants of diet quality. Regarding the finding that the IPR was not independently associated with diet quality, this finding is somewhat surprising given that diet quality is known to follow an SES gradient [33,34]. One mechanism through which low SES influences diet quality is food and nutrition insecurity [35]. In addition, education is also an indicator of SES [36] and was found to be significantly associated with the HEI-2020 score in the final model. Thus, it is possible that education and food insecurity accounted for the relationship between diet quality and income in the present analysis.

The determinants included in the final model predicted a higher proportion of variance in the HEI-2020 adequacy score than the moderation score. Interestingly, all the nonmodifiable determinants, including race and ethnicity, education, age group, and BMI category, were significantly associated with both adequacy and moderation score, whereas the only modifiable determinant associated with both scores was the use of nutrition labels. Number of meals consumed away from home, perceived diet quality, and food security status were associated with the adequacy score, but not the moderation score. The finding that food security status was associated with the adequacy score but not the moderation score is generally consistent with the literature, which suggests that individuals experiencing food insecurity typically reside in areas with lower access to affordable and healthy foods (i.e., adequacy components) [37].

In contrast, the findings that frequency of consuming food away from home and perceived diet quality were associated with the aggregated adequacy score but not the moderation score are not fully consistent with previous literature. Specifically, in previous waves of NHANES, individuals consuming food away from home ≤2 times a week had modestly higher scores for individual moderation and adequacy HEI-2020 components, including saturated fats and added sugars, greens and beans, total fruits, whole fruits, and whole grains relative to individuals consuming food away from home >2 times a week [11]. Regarding associations between perceived and measured diet quality, an analysis of young adults aged 18–30 y in the 2013–2014 NHANES wave showed that, for each 1-point increase in the HEI-2010 component scores for whole fruit [odds ratio (OR) = 1.098], whole grains (OR = 1.046), and empty calories (OR = 1.054), individuals had higher odds of having a higher perceived diet quality, whereas there were no associations between other component scores and perceived diet quality category [13].

These findings suggest that consuming food away from home and perceived diet quality are related to individual moderation and adequacy components of diet quality. This contrasts with the findings of the present study, where these determinants were not associated with the moderation score, which was derived from aggregating scores from the individual moderation components. Because determinants included in the present research were predictive of individual components within the moderation score but not associated with the aggregated score, this suggests future diet quality improvement interventions may benefit from developing targeted strategies for individual HEI-2020 components rather than broadly targeting adequacy or moderation constructs of diet quality.

Collectively, these findings suggest that to meaningfully improve total diet quality, different intervention strategies are likely needed for improving adequacy and moderation components, respectively. However, because the frequency of using the nutrition facts label to make food choices was associated with the largest increase in the total HEI-2020 score in the final model and was associated with adequacy and moderation component scores, strategies to increase use of nutrition labels warrant consideration in future research seeking to improve diet quality. Frequency of nutrition labels use follows both an educational and socioeconomic gradient, with regular users typically having more education and income than less frequent users [38], which suggests that limited nutrition literacy and income are barriers to regular use of nutrition labels. Although the cost of healthy foods is a critical and well-established barrier to consuming high-quality diets [39,40], the degree to which limited income and nutrition literacy independently influence use of nutrition labels for food purchasing and consumption is not clear. Thus, characterizing barriers and facilitators to nutrition label use across nutrition literacy and income spectrums may inform the development of interventions designed to increase the use of nutrition labels.

One intervention increasingly recognized as an effective strategy for promoting nutrition label use is front-of-package nutrition labels [41]. Front-of-package nutrition labels designed to interpret product healthfulness can improve consumers’ ability to identify healthier products, and can positively influence purchasing intentions for healthier products [42]. A randomized clinical trial conducted in United States adults showed that front-of-package labels using a spectrum design (i.e., labels rating products from least to most healthy) promoted healthier food choices in a simulated online shopping environment relative to other labeling schemes [43]. Notably, this finding was not moderated by income or education level, thus suggesting that spectrum front-of-package labels may reduce barriers to label use related to nutrition literacy. However, front-of-package labels may not be effective for influencing actual food consumption [42]. Further research is needed to examine how consumers use front-of-package nutrition labels and identify strategies to promote sustained use of these labels that result in improved diet quality.

These findings must be considered within the context of the study’s strengths and limitations. A strength of this article was that this study used a conceptual framework to identify relevant determinants of food choice captured by a nationally representative sample of United States adults. Thus, these results are likely generalizable to the noninstitutionalized adult civilian population in the United States. In terms of limitations, survey instruments used by NHANES were not designed to map onto the domains of food choice from the conceptual model used. As such, the determinants of food choice used in the present analysis do not fully represent each domain from the conceptual framework, and the food-internal factors domain was not represented in the present analysis. Furthermore, the present analysis was not designed to draw conclusions regarding the strength of associations between each domain in the conceptual model of food choice and diet quality. Future research could build on these findings by investigating which domains of food choice are most strongly associated with diet quality. Lastly, NHANES is a cross-sectional survey, which does not allow for conclusions to be drawn regarding the causal relationship between food choice determinants and diet quality.

In conclusion, consuming fewer meals away from home, having higher perceived diet quality, and using nutrition labels more frequently to make food choices were associated with moderately higher HEI-2020. These determinants of food choice predicted a higher proportion of variance in HEI-2020 adequacy components than moderation components. Taken together, these findings demonstrate the complexity of factors influencing diet quality and highlight the need for interventions to simultaneously target multiple determinants of food choice to improve diet quality.

## Author contributions

The authors’ responsibilities were as follows – KET: analyzed the data and drafted the manuscript; KSP: critically reviewed the manuscript and primarily responsible for final content; and both authors: read and approved the final manuscript.

## Data availability

Data described in the manuscript, codebook, and analytic code will be made available on request pending application and approval.

## Declaration of Generative AI and AI-assisted technologies in the writing process

The authors declare that no generative AI or AI-assisted technologies were used in the writing of this manuscript.

## Funding

KET was supported by the Department of Nutritional Sciences, Pennsylvania State University, and the National Center for Advancing Translational Sciences, National Institutes of Health, Grant TL1 TR002016. The content is solely the responsibility of the authors and does not necessarily represent the official views of the NIH. The funder had no role in design, analysis, data interpretation, or writing of the report.

## Conflict of interest

KSP is an Editorial Board Member for Current Developments in Nutrition and played no role in the Journal's evaluation of the manuscript. The authors report no conflicts of interest.
