# Supplementary material for: Associations between Determinants of Food Choice and the Healthy Eating Index-2020 in Adults: An NHANES 2017–March 2020 Analysis
Source: Curr Dev Nutr. 2026 Jun 12;10(7):109395. doi: 10.1016/j.cdnut.2026.109395 (PMC13355201; doi:10.1016/j.cdnut.2026.109395)
Supplement: Multimedia component 1 [file mmc1.docx]

# Supplemental Tables

## Supplemental Table 1: Multivariate associations between determinants of food choice and HEI-2020 adequacy components.^1^

| **HEI-2020 Adequacy Components** | | | | | | | | | | |
| --- | --- | --- | --- | --- | --- | --- | --- | --- | --- | --- |
|  | **Total Vegetables** | | **Greens and Beans** | **Total Fruit** | **Whole Fruits** | **Whole Grains** | **Total Dairy** | **Total Protein** | **Seafood and Plant Protein** | **Fatty Acids** |
| **Determinant** | | **---------------------------------------------------------------------------------------- β(SE)------------------------------------------------------------------------------------------** | | | | | | | | |
| **Age** |  | |  |  |  |  |  |  |  |  |
| 20 to 50 years | -0.07(0.07) | | -0.08(0.16) | -0.53(0.12)^†^ | -0.63(0.1)^#^ | -0.37(0.2) | 0.54(0.25)* | -0.05(0.04) | -0.44(0.18)* | -0.21(0.22) |
| 51 years and older | REF | | REF | REF | REF | REF | REF | REF | REF | REF |
| **Education** |  | |  |  |  |  |  |  |  |  |
| Less than 12th grade | -0.53(0.14)^†^ | | -0.41(0.25) | -0.46(0.22)* | -0.37(0.24) | -0.54(0.28) | 0.02(0.3) | -0.14(0.12) | -0.62(0.26)* | -0.92(0.44)* |
| 12th grade graduate | -0.50(0.13)^†^ | | -0.99(0.21)^#^ | -0.3(0.19) | -0.41(0.19)* | -0.82(0.3)* | -0.12(0.31) | -0.0030(0.11) | -0.72(0.18)^†^ | -0.25(0.3) |
| Some college/ Associates degree | -0.44(0.09)^#^ | | -0.75(0.16)^†^ | -0.52(0.14)* | -0.54(0.2)* | -0.59(0.25)* | 0.04(0.24) | 0.05(0.08) | -0.58(0.16)* | -0.27(0.28) |
| College graduate or above | REF | | REF | REF | REF | REF | REF | REF | REF | REF |
| **Race/ Ethnicity** |  | |  |  |  |  |  |  |  |  |
| Hispanic | 0.27(0.12)* | | 0.91(0.2)^†^ | 0.57(0.14)^†^ | 0.49(0.19)* | -0.68(0.13)^#^ | -1.04(0.19)^#^ | 0.23(0.06)^†^ | 0.74(0.18)^†^ | 0.71(0.25)* |
| Non-Hispanic Black | -0.17(0.14) | | 0.01(0.21) | 0.28(0.15) | -0.15(0.18) | -0.48(0.16)* | -1.73(0.16)^#^ | 0.03(0.06) | -0.02(0.11) | 1.53(0.25)^#^ |
| Other or Multiracial | 0.01(0.12) | | 0.3(0.21) | 0.45(0.11)^†^ | 0.29(0.16) | 0.12(0.21) | -0.98(0.23)^†^ | -0.03(0.08) | 0.32(0.25) | 0.98(0.27)* |
| Non-Hispanic White | REF | | REF | REF | REF | REF | REF | REF | REF | REF |
| **BMI Category** |  | |  |  |  |  |  |  |  |  |
| <18.5 kg/m² | -0.42(0.46) | | -0.82(0.48) | -0.66(0.36) | -1.29(0.4)* | -0.49(0.87) | 0.16(0.85) | 0.01(0.21) | 0.59(0.53) | -1.13(1.02) |
| 25 - <30 kg/m² | -0.05(0.17) | | -0.08(0.18) | -0.03(0.17) | 0.01(0.17) | -0.6(0.35) | 0.33(0.21) | 0.09(0.08) | 0.01(0.24) | -0.27(0.35) |
| ≥30 kg/m² | -0.13(0.16) | | -0.30(0.19) | -0.27(0.14) | -0.29(0.15) | -0.39(0.31) | 0.23(0.24) | 0.08(0.08) | 0.002(0.18) | -0.78(0.39) |
| 18.5 - <25 kg/m² | REF | | REF | REF | REF | REF | REF | REF | REF | REF |
| **Food Security Status** |  | |  |  |  |  |  |  |  |  |
| Very low food security | -0.4(0.14)* | | -0.26(0.17) | -0.46(0.17)* | -0.80(0.18)^†^ | -0.37(0.21) | 0.28(0.29) | -0.19(0.13) | -0.34(0.25) | -0.91(0.27)* |
| Low food security | -0.09(0.11) | | -0.09(0.19) | -0.1(0.14) | -0.09(0.19) | 0.03(0.21) | 0.06(0.22) | -0.15(0.08) | -0.01(0.19) | -0.15(0.22) |
| Marginal food security | -0.32(0.14)* | | 0.12(0.20) | 0.12(0.21) | 0.02(0.22) | -0.26(0.24) | 0.04(0.28) | -0.12(0.13) | -0.11(0.21) | -0.32(0.23) |
| Full food security | REF | | REF | REF | REF | REF | REF | REF | REF | REF |
| **Use of Nutrition Facts** |  | |  |  |  |  |  |  |  |  |
| Rarely/ never | -0.27(0.08)* | | -0.75(0.2)^†^ | -0.68(0.16)^†^ | -0.95(0.19)^#^ | -0.98(0.18)^#^ | -0.28(0.27) | -0.15(0.08) | -0.27(0.23) | -0.43(0.28) |
| Sometimes | -0.64(0.15)^†^ | | -0.47(0.18)* | -0.11(0.15) | -0.20(0.14) | -0.59(0.29)* | -0.03(0.28) | -0.08(0.04) | -0.12(0.18) | -0.51(0.24)* |
| Always/ Most of the time | REF | | REF | REF | REF | REF | REF | REF | REF | REF |
| **Perceived Diet Quality** |  | |  |  |  |  |  |  |  |  |
| Fair/poor | -0.45(0.12)^†^ | | -0.60(0.2)* | -0.52(0.18)* | -0.69(0.18)* | -0.55(0.25)* | -0.1(0.34) | 0.02(0.07) | -0.78(0.18)^†^ | -0.24(0.3) |
| Good | -0.18(0.13) | | -0.30(0.19) | -0.27(0.17) | -0.36(0.18) | -0.37(0.28) | 0.12(0.28) | 0.02(0.06) | -0.25(0.17) | -0.14(0.3) |
| Excellent/ Very good | REF | | REF | REF | REF | REF | REF | REF | REF | REF |
| **Frequency of Eating Out** |  | |  |  |  |  |  |  |  |  |
| ≥5 meals | -0.34(0.14)* | | -0.65(0.17)^†^ | -0.87(0.2)^†^ | -0.75(0.22)* | -1.06(0.33)* | -0.58(0.24)* | -0.06(0.08) | 0.21(0.18) | 0.60(0.24)* |
| 3-4 meals | -0.18(0.17) | | -0.69(0.2)* | -0.53(0.22)* | -0.44(0.24) | -0.78(0.33)* | -0.69(0.35) | 0.14(0.09) | -0.23(0.2) | 0.54(0.36) |
| 1-2 meals | -0.15(0.15) | | -0.74(0.17)^†^ | -0.35(0.15)* | -0.14(0.19) | -0.4(0.34) | 0.02(0.19) | 0.01(0.1) | 0.09(0.19) | -0.02(0.28) |
| 0 meals | REF | | REF | REF | REF | REF | REF | REF | REF | REF |
| **Model F Statistic** | 36.95^#^ | | 118.1^#^ | 101.76^#^ | 173.97^#^ | 97.35^#^ | 27.65^#^ | 19.26^#^ | 31.73^#^ | 19.91^#^ |
| **Adj R^2^** | 0.13 | | 0.14 | 0.14 | 0.17 | 0.12 | 0.07 | 0.03 | 0.09 | 0.06 |

## ^1^Table displays regression model effects and standard errors from nine models each specifying one HEI-2020 adequacy component score as the outcome variable, with modifiable (i.e., frequency of food away from home, perceived diet quality, use of nutrition facts, food security status) and non-modifiable (i.e., included age, race and ethnicity, BMI category, education) food choice determinants as the predictor variables.

## *p<0.05;^†^p<0.001; #p<0.0001

## Supplemental Table Two: Multivariate associations between determinants of food choice and HEI-2020 moderation components.

| **HEI-2020 Moderation Components** | | | | |
| --- | --- | --- | --- | --- |
|  | **Sodium** | **Saturated fat** | **Refined Gran** | **Added Sugar** |
| **Determinant** | **-------------------------------------------------------------------- β(SE)-------------------------------------------------------------------------------** | | | |
| **Age** |  |  |  |  |
| 20 to 50 years | -0.90(0.23)^†^ | -0.02(0.19) | -0.79(0.24)* | 0.07(0.18) |
| 51 years and older | REF | REF | REF | REF |
| **Education** |  |  |  |  |
| Less than 12th grade | 0.72(0.24)* | -0.18(0.41) | -0.52(0.51) | -1.32(0.3)^†^ |
| 12th grade graduate | 0.31(0.3) | 0.06(0.36) | -0.37(0.19) | -0.55(0.33) |
| Some college/ Associates degree | 0.07(0.22) | -0.4(0.26) | -0.06(0.24) | -0.74(0.17)^†^ |
| College graduate or above | REF | REF | REF | REF |
| **Race/ Ethnicity** |  |  |  |  |
| Hispanic | 0.26(0.21) | 0.94(0.21)^†^ | -1.42(0.25)^#^ | 0.53(0.21)* |
| Non-Hispanic Black | 0.45(0.23) | 0.95(0.23)^†^ | 0.2(0.25) | -0.32(0.26) |
| Other or Multiracial | -0.05(0.29) | 1.36(0.27)^#^ | -0.9(0.36)* | 0.24(0.24) |
| Non-Hispanic White | REF | REF | REF | REF |
| **BMI Category** |  |  |  |  |
| <18.5 kg/m² | -0.9(0.48) | -0.7(1.11) | -1.1(0.75) | 0.12(0.69) |
| 25 - <30 kg/m² | -0.4(0.34) | -0.53(0.31) | -0.18(0.37) | -0.05(0.29) |
| ≥30 kg/m² | -0.59(0.32) | -0.96(0.33)* | 0.14(0.3) | -0.35(0.25) |
| 18.5 - <25 kg/m² | REF | REF | REF | REF |
| **Food Security Status** |  |  |  |  |
| Very low food security | 0.51(0.31) | -0.56(0.33) | -0.04(0.38) | -1.02(0.26)^†^ |
| Low food security | -0.01(0.36) | 0.76(0.30)* | -0.37(0.3) | -0.42(0.25) |
| Marginal food security | 0.08(0.43) | 0.30(0.26) | -0.46(0.38) | -0.5(0.28) |
| Full food security | REF | REF | REF | REF |
| **Use of Nutrition Facts** |  |  |  |  |
| Rarely/ never | 0.33(0.29) | 0.002(0.27) | -0.27(0.31) | -1.66(0.24)^#^ |
| Sometimes | 0.18(0.29) | -0.19(0.22) | -0.57(0.26)* | -0.80(0.27)* |
| Always/ Most of the time | REF | REF | REF | REF |
| **Perceived Diet Quality** |  |  |  |  |
| Fair/poor | 0.37(0.35) | 0.03(0.34) | -0.82(0.26)* | -0.90(0.31)* |
| Good | -0.05(0.23) | 0.09(0.33) | -0.65(0.25)* | -0.58(0.21)* |
| Excellent/ Very good | REF | REF | REF | REF |
| **Frequency of Eating Out** |  |  |  |  |
| ≥5 meals | -0.43(0.38) | 0.10(0.4) | -0.35(0.31) | -0.71(0.24)* |
| 3-4 meals | -0.68(0.37) | -0.11(0.42) | -0.05(0.29) | -0.14(0.19) |
| 1-2 meals | -0.28(0.39) | -0.15(0.42) | -0.11(0.38) | -0.20(0.19) |
| 0 meals | REF | REF | REF | REF |
| **Model F Statistic** | 12.59^#^ | 23.38^#^ | 25.93^#^ | 684.26^#^ |
| **Adj R^2^** | 0.04 | 0.08 | 0.05 | 0.14 |

## ^1^Table displays regression model effects and standard errors from four models each specifying one HEI-2020 moderation component score as the outcome variable with modifiable (i.e., frequency of food away from home, perceived diet quality, use of nutrition facts, food security status) and non-modifiable (i.e., included age, race and ethnicity, BMI category, education) food choice determinants as the predictor variables.

## *p<0.05;^†^p<0.001; #p<0.0001
